# Advances in Gel-Based Electrolyte-Gated Flexible Visual Synapses for Neuromorphic Vision Systems

**DOI:** 10.3390/gels12040346

**Published:** 2026-04-21

**Authors:** Wanqi Duan, Yanyan Gong, Jinghai Li, Xichen Song, Zongying Wang, Qiaoming Zhang, Yuebin Xi

**Affiliations:** 1 State Key Laboratory of Green Papermaking and Resource Recycling, Qilu University of Technology (Shandong Academy of Sciences), Jinan 250353, Chinajinghaili@sdu.edu.cn (J.L.); 2 School of Chemistry and Chemical Engineering, Ministry of Education Key Laboratory of Special Functional Aggregated Materials, Shandong Key Laboratory of Advanced Organosilicon Materials and Technologies, Shandong University, Jinan 250100, China; 3 Shandong Provincial Key Laboratory for Science of Material Creation and Energy Conversion, Science Center for Material Creation and Energy Conversion, Shandong University, Qingdao 266237, China; 4 Comprehensive Technology Center of Suifenhe Customs District People’s Republic of China, Suifenhe 157399, China; songxc7140@163.com (X.S.); yingying_8985@126.com (Z.W.); 5 School of Physical Science and Technology, Southwest University, Chongqing 400715, China; zqm520@swu.edu.cn

**Keywords:** electrolyte-gated field effect transistor, gel electrolyte, flexible visual synapse, electric double layer, neuromorphic vision

## Abstract

Flexible electrolyte-gated synaptic field-effect transistors (EGFETs) have emerged as a promising platform for neuromorphic visual systems, owing to their low-voltage operation, diverse synaptic plasticity, and exceptional mechanical flexibility. In particular, gel-based electrolytes, including hydrogels and ion gels, play a pivotal role as functional gate dielectrics, enabling efficient ion transport and strong ion–electron coupling through electric double-layer (EDL) formation. By leveraging these unique properties at the semiconductor/gel interface, EGFETs can effectively emulate essential biological synaptic behaviors, including short-term and long-term plasticity under optical stimulation. The inherent compatibility of EGFETs with a broad range of semiconductor channels, gel electrolytes, and flexible substrates enables the development of wearable and conformable neuromorphic platforms that seamlessly integrate sensing, memory, and signal processing within a single device architecture. Recent advances in gel material engineering, such as polymer network design, ionic modulation, and nanofiller incorporation, have significantly improved ion transport dynamics, interfacial stability, and device performance. Despite remaining challenges related to ion migration stability, multi-physical field coupling, and large-area device uniformity, these developments have substantially advanced the practical potential of gel-based systems. This review provides a comprehensive overview of the operating mechanisms, gel-based material systems, synaptic functionalities, mechanical reliability, and future prospects of flexible electrolyte-gated visual synapses, highlighting their considerable potential for next-generation intelligent perception and artificial vision technologies.

## 1. Introduction

**Figure 1:**
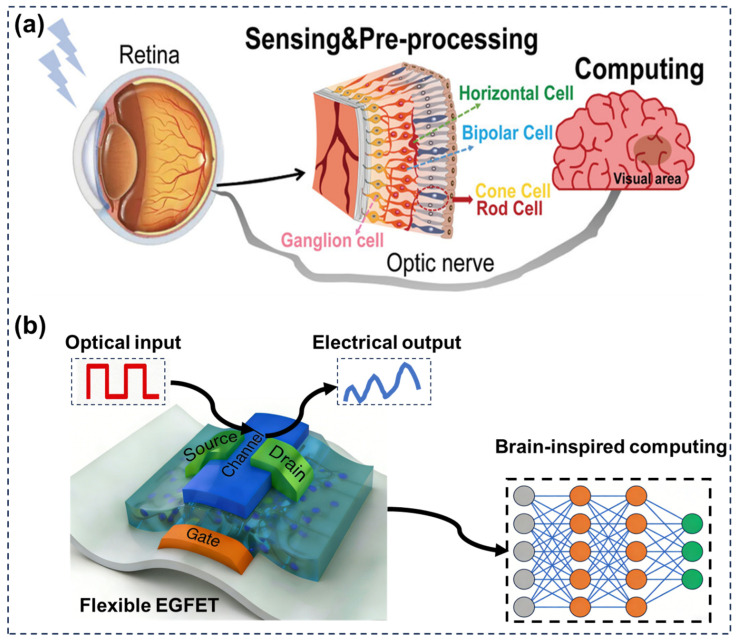
(**a**) Schematic illustration of the human visual perception system [5]. Copyright 2023, Wiley-VCH GmbH. (**b**) schematic diagram of flexible visual neuromorphic synaptic devices based on an EGFET.

The human visual system is an extraordinarily sophisticated and energy-efficient biological information-processing network, capable of performing photoreception, early-stage signal preprocessing, and adaptive learning through synaptic plasticity. In the retina, photoreceptors convert incident light into electrical signals while simultaneously filtering noise and extracting essential visual features, thereby optimizing information transmission to higher visual centers [1,2,3,4]. Synaptic plasticity further endows the visual system with learning and memory capabilities, enabling dynamic adaptation to complex and continuously changing environments (Figure 1a) [5]. In sharp contrast, conventional von Neumann computing architectures suffer from inherent limitations in visual information processing due to the physical separation of memory and processing units. This architectural bottleneck leads to frequent data transfer between distinct modules, resulting in excessive energy consumption, increased latency, and reduced efficiency, which severely hinder real-time and low-power visual computing [6,7,8]. To address these challenges, neuromorphic vision systems inspired by biological principles have emerged as a promising alternative, aiming to integrate sensing, processing, and memory functions within unified hardware platforms [9,10].

Artificial visual synapses are key building blocks of neuromorphic vision systems, designed to emulate essential synaptic functions such as signal transmission modulation and plasticity associated with learning and memory [11]. Among various device concepts, resistive switching and memristive devices have been extensively explored owing to their simple structures and high scalability [12]. However, these devices often face challenges related to device-to-device variability, limited tunability, nonlinear conductance updates, and vulnerable stability [13]. In contrast, transistor-based synaptic devices offer distinct advantages due to their multi-terminal architectures, which enable precise, continuous, and reversible modulation of synaptic weights, as well as improved operational stability and reproducibility [14,15,16]. These features make transistor-type synapses particularly attractive for implementing complex neuromorphic functions and advanced signal processing in artificial vision systems.

Among transistor-based synaptic devices, electrolyte-gated field-effect transistors (EGFETs) have attracted increasing attention because of their unique operating mechanisms and excellent compatibility with flexible electronics [17]. Employing an electrolyte gate dielectric allows EGFETs to operate at ultra-low voltages through electric double layer formation, substantially lowering power consumption for wearable and implantable neuromorphic applications (Figure 1b) [18]. Gel-based electrolytes, including hydrogels and ion gels, have emerged as key functional materials in flexible electrolyte-gated devices, fundamentally influencing the performance of EGFETs. Compared with conventional solid dielectrics, they offer distinct advantages such as high ionic conductivity, mechanical softness, and excellent interfacial conformability. The three-dimensional polymer network within gels provides continuous ion transport pathways, facilitating efficient electric double layer (EDL) formation at low operating voltages. Moreover, the tunable physicochemical properties of gels through crosslinking density, ionic composition, and nanofiller incorporation enable precise control over ion dynamics and device performance. These characteristics make gel electrolytes particularly suitable for constructing flexible, bio-compatible, and high-performance neuromorphic synaptic devices. The strong ionic-electronic coupling in EGFETs enables dynamic and history-dependent modulation of channel conductance, closely mimicking biological synaptic behaviors such as short-term plasticity (STP), long-term potentiation (LTP), and depression [19]. Moreover, EGFETs inherently exhibit outstanding mechanical flexibility and biocompatibility, allowing seamless integration with flexible substrates and bio-interfacing systems [20]. As a result, flexible visual neuromorphic synaptic devices based on gel-based EGFETs provide a compelling platform that bridges biological vision principles with advanced electronic functionalities, offering new opportunities for next-generation intelligent vision technologies [16]. Despite the rapid progress in neuromorphic vision devices, existing review articles have primarily focused on either material platforms (e.g., perovskites, 2D semiconductors) or device architectures such as memristors and photodetectors, with limited attention to electrolyte-gated transistor systems, particularly those based on gel electrolytes. Moreover, the critical role of ion–electronic coupling, interfacial EDL formation, and ion transport dynamics in governing synaptic behaviors has not been systematically addressed. In addition, the integration of flexibility, biocompatibility, and visual sensing functionalities remains insufficiently explored in prior reviews [21].

In this review, we specifically focus on gel-based electrolyte-gated flexible visual synapses and provide a comprehensive overview from the perspectives of device physics, material design, and system-level functionality. We elucidate the operating mechanisms of EGFETs, emphasizing ion migration, EDL formation, and their coupling with electronic transport. We then summarize recent advances in gel electrolyte materials, including hydrogels and ion gels, highlighting strategies such as polymer network engineering, ionic modulation, and nanofiller incorporation. Furthermore, we discuss synaptic functionalities with particular emphasis on the physical origins of STP and LTP. Specifically, STP arises from transient ion accumulation and rapid relaxation processes, leading to volatile conductance changes, whereas LTP is associated with sustained ion redistribution, deep trap states, or structural rearrangements within the gel or at the interface, resulting in non-volatile or long-retention memory behavior. Finally, we address mechanical reliability, emerging applications in neuromorphic vision systems, and key challenges toward practical implementation.

## 2. Working Principles of Electrolyte-Gated Synaptic Transistors

EGFETs have emerged as a powerful platform for neuromorphic electronics owing to their low-voltage operation, high interfacial capacitance, and strong coupling between ionic and electronic degrees of freedom. In synaptic transistors, gel-based electrolytes such as hydrogels and ion gels play a central role by enabling ion migration and EDL formation at the electrolyte/semiconductor interface, thereby modulating the channel conductance and emulating essential synaptic functions, including STP, long-term LTP, and learning-forgetting behaviors [22,23]. When integrated with photosensitive semiconductors, EGFETs enable intrinsic optical–electrical coupling through the interplay between photogenerated carriers and ionic dynamics. Specifically, incident light generates electron–hole pairs in the semiconductor channel or heterojunction, leading to a redistribution of local electric fields. This process drives ion migration within the gel electrolyte and modulates the EDL at the interface, resulting in a persistent or transient change in channel conductance that resembles synaptic weight modulation. The temporal dynamics of ion relaxation and charge trapping further give rise to characteristic synaptic behaviors such as EPSC, PPF, and long-term memory effects under optical stimulation. Compared with conventional photodetector-based architectures that require separate sensing, memory, and processing units, such electrolyte-gated phototransistors inherently integrate light perception and synaptic computation within a single device. This in-sensor computing capability significantly reduces data transfer latency and power consumption while enabling spatiotemporal signal processing analogous to biological visual systems. As a result, EGFET-based optoelectronic synapses offer a highly efficient and compact platform for next-generation neuromorphic vision applications.

### 2.1. EDL Formation

**Figure 2:**
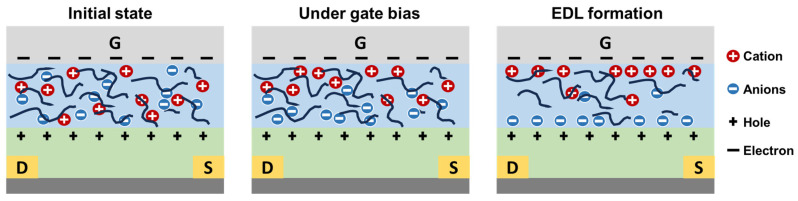
Schematic illustration of ion–electron coupling in gel-based electrolyte-gated transistors.

A defining feature of EGFETs is the formation of an EDL at the electrolyte/semiconductor and electrolyte/gate interfaces, as schematically illustrated in Figure 2. Upon application of a gate voltage (V_G_), mobile ions within the electrolyte migrate toward interfaces of opposite polarity, leading to the formation of two nanometer-scale charge layers: one consisting of accumulated ions in the electrolyte and the other composed of induced electronic charges in the semiconductor channel [24]. This interfacial EDL functions as an ultra-high-capacitance gate dielectric, typically reaching values of several µF cm^−2^, which are orders of magnitude higher than those of conventional solid-state dielectrics [25,26]. In gel electrolytes, the formation of EDLs is further influenced by the three-dimensional polymer network structure, which provides continuous ion transport pathways and facilitates ion accumulation at the interface. The presence of polar functional groups within the gel matrix has been reported to facilitate ion dissociation and stabilize interfacial charge accumulation, thereby enhancing the EDL capacitance and dynamic response of EGFETs [27,28].

As illustrated in Figure 2, the operation of gel-based electrolyte-gated transistors is governed by ion–electron coupling mediated by the formation of EDLs. In the absence of gate bias, mobile cations and anions are uniformly distributed within the gel electrolyte, which consists of an interconnected three-dimensional polymer network that provides continuous ion transport pathways. Upon application of a gate voltage, ions migrate through this polymer matrix along these pathways, driven by the electric field, leading to dynamic charge redistribution within the electrolyte. For ion-impermeable semiconductors, ions are confined at the electrolyte/semiconductor interface, where they accumulate to form an EDL that induces a strong local electric field. This field efficiently modulates the carrier density at the channel surface via purely electrostatic coupling, without ion penetration into the semiconductor. In contrast, for ion-permeable semiconductors, mobile ions can partially penetrate into the bulk under gate bias, resulting in a combined electrostatic and electrochemical modulation of channel conductance. The exceptionally large EDL capacitance enables effective carrier density modulation at very low operating voltages, often below 1 V, which is crucial for energy-efficient neuromorphic computing and flexible electronics [29]. Importantly, EDL formation is inherently a dynamic process governed by ion mobility, electrolyte composition, temperature, and device geometry. Consequently, the temporal evolution of ion redistribution gives rise to time-dependent conductance modulation, closely resembling the transient response characteristics of biological synapses [30]. In synaptic transistors, the migration and accumulation of ions under gate bias directly control the accumulation or depletion of charge carriers in the channel. Cations or anions attracted to the semiconductor surface generate a strong local electric field, modulating channel conductance without necessarily involving chemical reactions [31]. This electrostatically driven process forms the physical basis for volatile synaptic behaviors and fast signal processing in EGFET-based neuromorphic devices [32,33,34].

### 2.2. Volatile vs. Non-Volatile Synaptic Modulation

Depending on the nature of ion-channel interaction, EGFETs can exhibit either volatile or non-volatile conductance changes, corresponding to short-term and long-term synaptic plasticity, respectively. Volatile synaptic modulation, typically associated with STP, arises from reversible surface charge modulation induced by EDL formation. In gel-based systems, this process is strongly dependent on ion mobility within the polymer network and the relaxation dynamics of the gel matrix, which govern the temporal characteristics of STP. In this case, ions accumulate at the electrolyte/semiconductor interface under an external stimulus and relax back to their equilibrium positions once the stimulus is removed. The resulting conductance change decays over time, mimicking biological phenomena such as paired-pulse facilitation (PPF), paired-pulse depression (PPD), and short-term memory. STP is particularly sensitive to pulse amplitude, duration, and frequency, reflecting the kinetics of ion migration and relaxation.

In contrast, non-volatile synaptic modulation, corresponding to LTP, involves more persistent ionic processes, such as ion penetration into the semiconductor bulk, ion trapping at defect sites, or electrochemical redox reactions [35,36]. In these cases, repeated or stronger stimuli can drive ions to overcome energy barriers and become embedded within the channel or interfacial regions, leading to stable conductance changes that persist even after removal of the gate bias. This mechanism enables the emulation of long-term memory, learning saturation, and memory consolidation. The coexistence of volatile and non-volatile behaviors in EGFETs provides a versatile platform for mimicking the hierarchical memory functions of biological synapses. By tailoring electrolyte composition, channel material, and device architecture, it is possible to finely tune the balance between STP and LTP, enabling adaptive and multifunctional neuromorphic systems.

### 2.3. Optical–Electrical Coupling in Visual Synapses

For visual neuromorphic applications, EGFETs are often integrated with photosensitive semiconductor channels, allowing optical stimuli to directly modulate synaptic behavior. Upon light illumination, photogenerated electron–hole pairs are created in the semiconductor, altering the channel conductance and interacting with the ionic gating process [32,37]. The coupling between photocarrier generation and ionic motion gives rise to a rich set of optoelectronic synaptic behaviors. First, photogenerated carriers can be accumulated or depleted by the EDL field, effectively amplifying the photoresponse under low V_G_. Second, illumination can modify the local electric field distribution and ion dynamics, leading to light-dependent synaptic plasticity. For example, light pulses can act as presynaptic stimuli, inducing excitatory or inhibitory postsynaptic currents analogous to biological visual synapses [38,39]. In gel-based EGFETs, the coupling between photocarrier generation and ionic motion is further modulated by the viscoelastic properties and ionic transport characteristics of the gel electrolyte, enabling tunable optoelectronic synaptic responses.

More importantly, light-ion–electron synergistic modulation enables unique functionalities that are difficult to achieve with purely electrical stimuli. Photogenerated carriers can assist ion migration, release trapped ions, or stabilize ionic distributions, thereby extending the retention time of synaptic states [22,40]. This synergistic effect allows visual synaptic devices to exhibit both fast optical sensing and adaptive memory functions, such as light-tunable STP/LTP transition, optical learning, and image preprocessing. In flexible visual neuromorphic systems, this optical–electrical coupling is particularly advantageous, as it enables simultaneous sensing, memory, and computation within a single device [41]. By leveraging the intrinsic dynamics of EDL formation, ionic motion, and photocarrier generation, EGFETs provide a biologically inspired and hardware-efficient route toward next-generation artificial vision systems [42,43]. Overall, the interplay between gel structure, ion dynamics, and electronic transport fundamentally determines the synaptic performance of EGFETs, highlighting the critical role of gel materials in the design of next-generation neuromorphic devices.

## 3. Material Systems for Flexible Electrolyte-Gated Visual Synapses

To realize flexible electrolyte-gated visual synaptic devices with multifunctional sensing and memory capabilities, material selection across the semiconductor channel, electrolyte, and flexible substrates/electrodes is of central importance. Table 1 summarizes representative material systems and their associated synaptic functions reported in recent studies, highlighting the diversity and tunability of electrolyte-gated neuromorphic platforms.

### 3.1. Semiconductor Channel Materials

The choice of semiconductor channel materials critically determines the electrical performance, mechanical compliance, and optical functionality of EGFETs [55,56]. As summarized in Table 1, a wide range of organic, inorganic, and low-dimensional semiconductors have been explored to emulate synaptic behaviors such as PPF, STP, and LTP. Organic semiconductors, particularly conjugated polymers such as poly(3-hexylthiophene) (P_3_HT) and donor-acceptor small molecules like dinaphtho [2,3-b:2′,3′-f]thieno [3,2-b]thiophene (DNTT), have been extensively investigated owing to their solution processability, intrinsic mechanical softness, and compatibility with flexible substrates. For example, P_3_HT-based EGFETs combined with ion-gel dielectrics have demonstrated reliable STP, PPF, and even LTP behaviors on elastomeric substrates such as PDMS [44,54]. Similarly, DNTT channels gated by acidic polyelectrolytes or dextran electrolytes enable controllable transitions between STP and LTP, highlighting the strong coupling between organic semiconductors and ionic dielectrics [45,46]. These characteristics make organic semiconductors particularly suitable for highly flexible, wearable, and bio-integrated neuromorphic systems, although their relatively low carrier mobility and environmental instability may limit high-speed operation and long-term reliability.

In parallel, inorganic oxide semiconductors, including indium gallium zinc oxide (IGZO), indium zinc tin oxide (InZnSnO), and SnO_2_, offer complementary advantages such as higher carrier mobility, superior environmental stability, and optical transparency. These features are particularly advantageous for visual synaptic devices requiring simultaneous light sensing and signal processing. As shown in Table 1, IGZO-based EGFETs demonstrate a wide range of synaptic functionalities, including STP, LTP, PPF, and PPD, depending on the choice of electrolyte and device configuration [29,49]. Oxide nanowire systems, such as InZnSnO nanowires, further extend the material landscape by enabling low-dimensional transport and enhanced ion-channel coupling [53]. Compared with organic counterparts, oxide semiconductors are more suitable for applications requiring high electrical performance, operational stability, and optical transparency, such as integrated optoelectronic neuromorphic systems, although their mechanical flexibility is generally inferior. Two-dimensional and nanostructured materials, including MoS_2_, MXene, and single-walled carbon nanotubes (SWCNTs), represent another important class of channel materials. Their large surface-to-volume ratios facilitate strong electrostatic and ionic interactions under electrolyte gating, enabling efficient synaptic modulation at low operating voltages. As summarized in Table 1, MoS_2_-based devices gated by polymer electrolytes or ionic liquids exhibit a combination of STP, LTP, and PPF behaviors, underscoring the versatility of low-dimensional semiconductors for neuromorphic vision applications [30,51]. These low-dimensional materials are particularly advantageous for low-power and high-sensitivity applications due to their strong interfacial coupling, although challenges such as material uniformity, large-area fabrication, and device reproducibility remain. Overall, the diverse semiconductor material systems reported to date provide a broad design space for tailoring electrical, optical, and mechanical properties to meet the specific requirements of flexible visual neuromorphic synapses. In general, organic semiconductors favor flexibility and biocompatibility, oxide semiconductors excel in stability and performance, while low-dimensional materials offer superior interfacial sensitivity and low-voltage operation, highlighting the importance of material selection based on specific application scenarios.

### 3.2. Gel Electrolytes and Ionic Dielectric Materials

Electrolytes play a central role in EGFETs by enabling ionic transport and the formation of EDLs at the electrolyte/semiconductor interface. Among various electrolyte systems, gel-based electrolytes, including ion gels and hydrogels, have become the dominant material platforms for flexible synaptic transistors. These gel systems combine the advantages of liquid electrolytes (high ionic conductivity) and solid-state materials (mechanical stability), making them particularly suitable for flexible and wearable neuromorphic devices. Electrolytes play a central role in EGFETs by enabling ionic transport and the formation of EDLs at the electrolyte/semiconductor interface. As illustrated in Table 1, various electrolyte systems including ion gels, polymer electrolytes, ionic liquids, and bio-derived polyelectrolytes have been employed to regulate synaptic behavior. Ion gels, typically composed of ionic liquids immobilized within polymer matrices, are among the most widely adopted electrolytes due to their high ionic conductivity, wide electrochemical stability window, and excellent mechanical flexibility. These properties generally result in higher capacitance, faster response, and improved operational stability compared to other gel systems, enabling efficient EDL formation at ultra-low gate voltages and supporting robust synaptic functions such as STP, PPF, and LTP in both organic and inorganic semiconductor systems [44,54]. However, their relatively complex fabrication processes and potential ion leakage or encapsulation challenges may limit large-area scalability and long-term reliability. Polymer electrolytes, such as LiClO_4_-doped polyethylene oxide (PEO) or PMMA-based electrolytes, offer good film-forming capability and chemical tunability. Devices employing these electrolytes have demonstrated reproducible STP and LTP behaviors in MoS_2_, In_2_O_3_, and oxide nanowire channels, highlighting their suitability for flexible and large-area neuromorphic platforms [24,30,52,53]. Nevertheless, their ionic conductivity is typically lower than that of ion gels, which may lead to slower switching speed and reduced synaptic response efficiency.

Beyond synthetic electrolytes, bio-derived and natural polymer electrolytes including dextran, konjac glucomannan, and acidic polyelectrolytes have attracted increasing attention. These materials combine ionic conductivity with biodegradability, softness, and biocompatibility, enabling environmentally friendly and bio-interfaced synaptic devices. As summarized in Table 1, such electrolytes can support both volatile and non-volatile synaptic modulation in organic and oxide semiconductor channels [29,45,46]. However, their performance is often sensitive to environmental conditions (e.g., humidity and temperature), and their long-term stability and reproducibility remain key challenges. Collectively, electrolyte selection critically influences ion mobility, operating voltage, response speed, mechanical durability, and long-term stability, thereby dictating the overall synaptic performance of flexible visual neuromorphic devices.

In gel electrolytes, the three-dimensional polymer network structure plays a critical role in regulating ion transport and device performance. The interconnected porous framework provides continuous pathways for ion migration, while functional groups such as hydroxyl, carboxyl, and ether groups facilitate ion dissociation and transport through strong ion-dipole interactions. Furthermore, nanofiller incorporation (e.g., cellulose nanofibers, silica nanoparticles, or MXene nanosheets) has emerged as an effective strategy to enhance ionic conductivity, mechanical strength, and interfacial stability. These nanostructured additives can construct hierarchical ion transport channels and suppress ion aggregation, thereby improving the dynamic response and long-term stability of synaptic devices. Despite these advantages, gel electrolytes still face several inherent limitations, including ion migration-induced hysteresis, relatively slow response speed compared to solid-state dielectrics, and a trade-off between mechanical softness and ionic conductivity. Therefore, rational design of gel electrolytes, including network structure optimization, ionic modulation, and multifunctional additive incorporation, is essential for achieving high-performance, stable, and flexible neuromorphic synaptic devices.

### 3.3. Flexible Substrates and Electrodes

Flexible substrates and electrodes provide the mechanical foundation for wearable and deformable neuromorphic vision systems. As shown in Table 1, a variety of polymer substrates including polyimide (PI), polyethylene terephthalate (PET), polyethylene naphthalate (PEN) and PDMS have been employed to support EGFETs under bending and deformation. For electrodes, transparent conductive materials such as indium tin oxide (ITO) remain widely used due to their high optical transparency and compatibility with visual sensing. However, their limited mechanical durability under strain has motivated the exploration of alternative electrode materials. Silver nanowire (AgNW) networks, PEDOT:PSS, carbon nanotubes, and graphene-based electrodes have been successfully integrated with flexible substrates to achieve improved mechanical compliance while maintaining excellent electrical performance [50,54]. The synergistic integration of flexible substrates, compliant electrodes, and EGFETs enables stable synaptic functionality under mechanical deformation. This integration is essential for the development of next-generation wearable, implantable, and bio-integrated neuromorphic vision systems, where mechanical adaptability and functional reliability are equally critical.

## 4. Flexible Visual Synaptic Functions Enabled by EGFETs

### 4.1. Optical-Stimulated Synaptic Plasticity

EGFETs exhibit a rich repertoire of synaptic plasticity behaviors under optical stimulation, enabling close emulation of key functionalities of biological synapses. Upon illumination with optical pulses, photogenerated charge carriers in the semiconductor channel interact synergistically with mobile ions in the electrolyte, resulting in dynamic modulation of channel conductance. This process gives rise to excitatory and inhibitory postsynaptic currents (EPSCs and IPSCs), which serve as electronic analogues of synaptic excitation and inhibition in biological neural networks [57,58]. In gel-based EGFETs, this optoelectronic synaptic behavior is strongly influenced by the ionic transport characteristics and viscoelastic properties of the gel electrolyte, which regulate ion accumulation, relaxation dynamics, and interfacial charge coupling.

**Figure 3:**
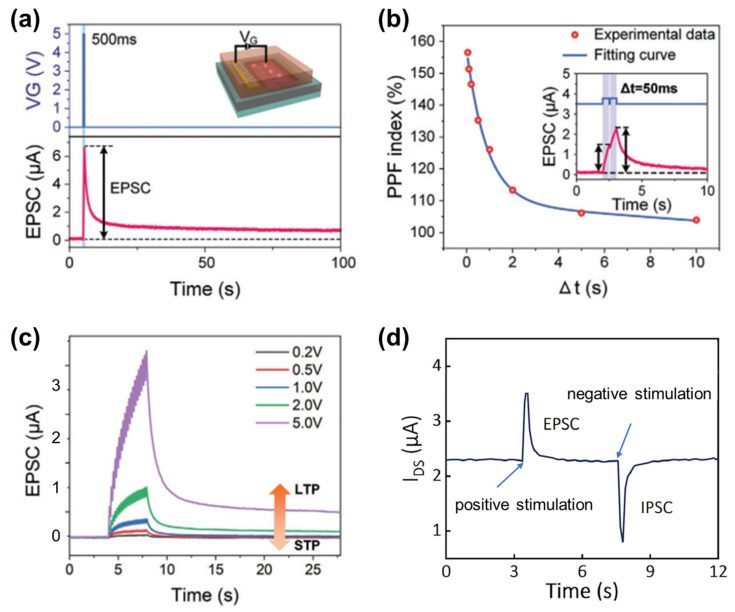
(**a**) EPSC induced by an electrical pulse. (**b**) PPF index as a function of the inter-pulse interval. (**c**) Transition from STP to LTP in the synaptic transistor achieved by increasing the pulse amplitude [49]. Copyright 2025, Wiley-VCH GmbH. (**d**) Inhibitory postsynaptic current (IPSC) and EPSC obtained under different V_G_.

As typically observed, a single optical or electrical gate pulse can induce a pronounced transient EPSC followed by a gradual decay, reflecting the combined effects of carrier generation, ion accumulation at the interface, and subsequent relaxation processes (Figure 3a) [49]. The temporal evolution of EPSC provides direct insight into synaptic response dynamics and forms the basis for STP. In particular, PPF characterized by an enhanced second EPSC in response to two closely spaced stimuli, emerges from the incomplete relaxation of ions and carriers between successive pulses. The dependence of the PPF index on the inter-pulse interval (Δt) highlights the intrinsic time constants of ionic migration and recombination processes, enabling tunable temporal filtering and signal integration (Figure 3b). The PPF behavior in gel-based systems is closely related to the ion diffusion and relaxation processes within the polymer network, which define the temporal response window of synaptic modulation. Beyond transient responses, the strength and persistence of synaptic modulation can be continuously tuned by adjusting stimulation conditions. As illustrated by the gradual transition from STP to LTP, increasing the pulse amplitude or repetition strength drives more substantial ionic redistribution and interfacial charge trapping, leading to progressively enhanced and longer-lasting conductance states (Figure 3c). This controllable STP-to-LTP transition closely mimics biological learning processes, in which repeated or stronger stimuli consolidate short-term memory into long-term memory.

In addition to excitatory behavior, EGFET-based synaptic devices are capable of emulating inhibitory synaptic functions. By modulating the polarity and magnitude of the gate bias, both EPSC and IPSC responses can be selectively induced within a single device (Figure 3d) [53]. This bidirectional synaptic modulation is essential for implementing balanced excitation–inhibition dynamics, which are fundamental to stable and energy-efficient neural computation. Collectively, these optically stimulated synaptic plasticity behaviors, including EPSC and IPSC generation, PPF, STP, and LTP, establish EGFETs as a powerful platform for neuromorphic vision systems. Their capability to convert visual stimuli into adaptive synaptic signals with high temporal resolution and tunable memory characteristics enables efficient preprocessing of visual information directly at the device level. In this way, sensing, memory, and computation are intrinsically integrated within a unified hardware framework.

### 4.2. Visual Learning and Memory Behaviors

**Figure 4:**
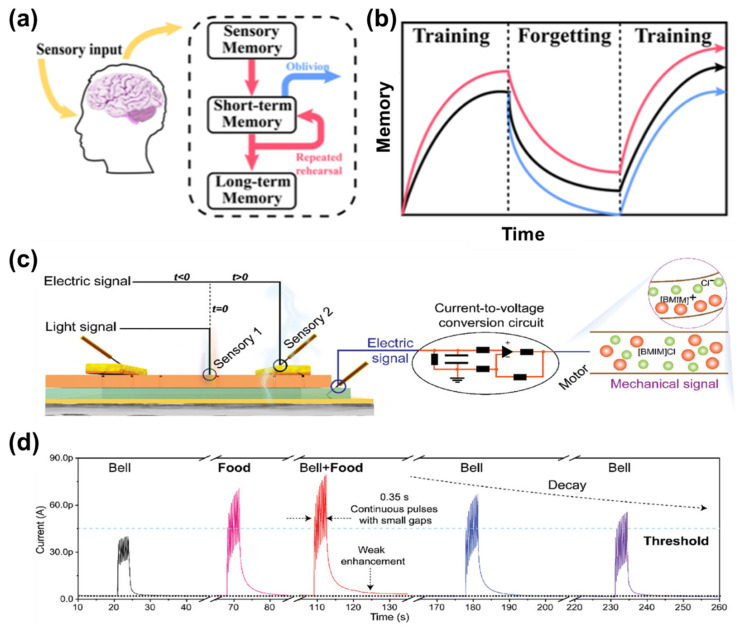
(**a**) Schematic illustration of the human memory process, including sensory memory, short-term memory, and long-term memory. (**b**) Schematic representation of the learning-forgetting-memory processes [29]. Copyright 2025, American Chemical Society. (**c**) Schematic illustration of an artificial afferent/efferent nerve system, in which electro-optical perovskite sensing and processing elements are coupled with artificial muscles to transduce signals from receptors to motor neurons. (**d**) Emulation of classical conditioning learning using an OHP-based artificial synapse. Electrical and optical stimuli (light intensity: 0.72 mW cm^−2^) are employed as the unconditioned (food) and conditioned (bell) stimuli, respectively. The number of pulses is 10 and the electrical and optical pulse duration is 0.35 s [48]. Copyright 2023, Wiley-VCH GmbH.

Flexible EGFET-based visual synapses are capable of implementing learning and memory behaviors that closely resemble those of biological neural systems [59]. In biological cognition, sensory inputs are first registered as transient sensory memory, which can evolve into short-term memory (STM) and, upon repeated reinforcement, further consolidate into long-term memory (LTM, Figure 4a) [29]. Analogously, in electrolyte-gated visual synapses, the synaptic weight, typically represented by the channel conductance or the postsynaptic current, exhibits gradual accumulation under repeated optical stimulation and spontaneous decay in the absence of stimuli, thereby reproducing key characteristics of biological learning and forgetting processes. The evolution of synaptic weight under training and forgetting cycles can be described by characteristic conductance growth and decay curves (Figure 4b). During the training phase, repeated optical pulses induce progressive conductance enhancement, reflecting the accumulation of photogenerated carriers and the gradual redistribution of mobile ions at the electrolyte/semiconductor interface. In particular, in gel electrolytes, the three-dimensional polymer network provides confined yet continuous ion transport pathways, enabling gradual and controllable ionic redistribution that is essential for stable learning and memory behaviors. When stimulation is removed, the conductance decays over time due to ionic relaxation and carrier recombination, corresponding to memory fading. Notably, retraining can effectively restore and even further enhance the synaptic weight, demonstrating learning reinforcement and robust memory retention, which are key characteristics of adaptive learning systems.

Beyond individual synaptic responses, EGFET-based devices can be integrated into artificial sensory-motor pathways that mimic biological afferent and efferent nerve systems (Figure 4c) [48]. In such architectures, optical signals are detected and processed by optoelectronic synaptic elements, converted into electrical signals, and subsequently transmitted to actuators or artificial muscles. This signal transduction pathway enables the direct coupling of visual perception, synaptic processing, and motor response, highlighting the potential of flexible EGFETs for embodied neuromorphic systems and bio-inspired robotics. At the behavioral level, electrolyte-gated visual synapses are capable of emulating higher-order learning phenomena, such as classical conditioning. As illustrated in Figure 4d, electrical and optical stimuli can be assigned as unconditioned (food) and conditioned (bell) signals, respectively. Through repeated paired stimulation, an initially ineffective conditioned stimulus gradually induces a pronounced synaptic response, indicating successful associative learning. The persistence and decay of this conditioned response further reflect memory formation, consolidation, and forgetting processes in the artificial synapse. Importantly, the degree and retention time of synaptic weight modulation are strongly dependent on the intensity, duration, and frequency of optical inputs. Stronger or more frequent stimulation promotes the transition from STM to LTM by driving more substantial ionic redistribution or interfacial charge trapping, leading to longer-lasting conductance states. This stimulus-dependent plasticity endows flexible EGFET-based visual synapses with the ability to adaptively encode visual information according to environmental conditions, providing a solid hardware foundation for adaptive learning, memory consolidation, and experience-dependent response tuning in flexible neuromorphic vision systems. The transition from STM to LTM in gel-based systems is often governed by ion trapping, network relaxation, and structural reconfiguration within the gel matrix, which contribute to long-term conductance retention.

### 4.3. Image Sensing and In-Sensor Preprocessing

A key advantage of EGFET-based flexible visual synaptic devices lies in their intrinsic ability to integrate image sensing and preprocessing within a single device architecture. Unlike conventional vision systems that rely on separate photodetectors, memory units, and processing modules, EGFETs-based synaptic devices directly convert optical stimuli into modulated synaptic currents. This direct transduction inherently couples photodetection with synaptic weight modulation, enabling in-sensor computing in which visual information is processed at the point of data acquisition [60,61]. In gel-based EGFET arrays, the spatiotemporal dynamics of ion migration within the gel electrolyte play a critical role in enabling analog signal processing, such as temporal filtering and noise suppression.

**Figure 5:**
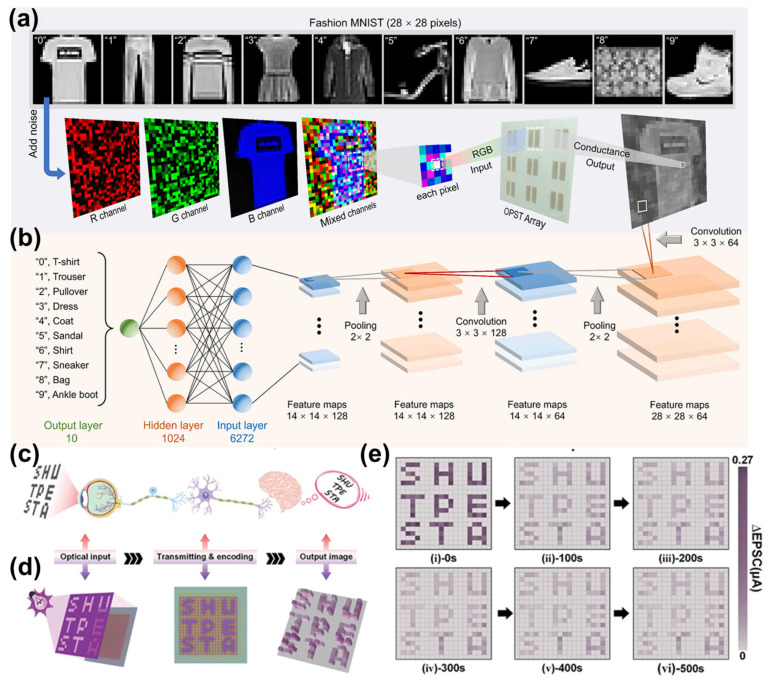
Artificial visual system based on optical synaptic transistor enabled image preprocessing and CNN-based recognition. (**a**) Three types of images: original Fashion-MNIST images, images corrupted with red and green Gaussian noise, and denoised images after preprocessing by optical synaptic transistors. (**b**) Schematic illustration of the CNN architecture, consisting of two convolutional layers (3 × 3 kernels), two pooling layers, and a fully connected layer for classification [9]. Copyright 2025, Wiley-VCH GmbH. (**c**) Schematic illustration of the human visual system. (**d**) Schematic diagram of the artificial vision system based on a EGFET array. (**e**) EPSC maps of a 20 × 20 EGFET array recorded at 0, 100, 200, 300, 400, and 500 s after the termination of optical stimulation [49]. Copyright 2025, Wiley-VCH GmbH.

As illustrated by representative image-processing schemes (Figure 5a), optical synaptic transistor arrays can directly operate on pixel-level optical inputs, such as Fashion-MNIST images corrupted by multi-channel Gaussian noise [9]. Through the intrinsic temporal dynamics of EPSCs, noisy optical inputs are selectively suppressed while salient image features are retained, resulting in effective denoising prior to digital processing. This device-level preprocessing exploits the natural decay and integration behavior of synaptic currents, eliminating the need for complex algorithmic filtering at the software level. The preprocessed synaptic outputs can be seamlessly interfaced with conventional machine learning architectures for higher-level recognition tasks. For example, compared with raw noisy inputs, the use of synaptic preprocessing can enhance classification accuracy by 10–30% under high-noise conditions, while also reducing the need for complex digital filtering algorithms [62]. As shown in Figure 5b, denoised images are fed into convolutional neural networks (CNNs), enabling more accurate and robust classification. In addition to accuracy improvement, this hybrid hardware-software framework reduces data redundancy and computational load, as a substantial portion of noise filtering and feature extraction is performed directly at the device level. This leads to lower energy consumption and faster processing compared to conventional vision systems, where all raw data must be transmitted and processed digitally.

Importantly, the operating principle of such artificial vision systems closely parallels that of the human visual pathway. In biological vision, early-stage processing in the retina and visual cortex performs essential feature extraction and noise filtering before higher-level cognitive interpretation (Figure 5c) [49]. Similarly, artificial vision systems based on EGFET arrays integrate sensing, memory, and preprocessing at the hardware level (Figure 5d), effectively mimicking the hierarchical organization of biological visual perception. The spatiotemporal evolution of synaptic responses within device arrays further enables dynamic visual information processing. As shown by time-dependent EPSC maps of a EGFET array (Figure 5e), optical stimulation induces spatially resolved conductance patterns that gradually decay over time, providing a hardware-based mechanism for temporal integration and short-term visual memory. Such behavior is particularly advantageous for processing dynamic visual scenes, motion detection, and event-driven vision. By unifying image sensing, memory, and preprocessing within flexible, low-voltage devices, EGFET-based visual synapses significantly reduce data redundancy and energy consumption associated with conventional vision systems. Their mechanical flexibility, low-power operation, and bio-inspired functionality position them as a promising platform for next-generation neuromorphic vision technologies, particularly in wearable, implantable, and bio-integrated applications.

## 5. Mechanical Flexibility and Biodegradable Neuromorphic Devices

**Figure 6:**
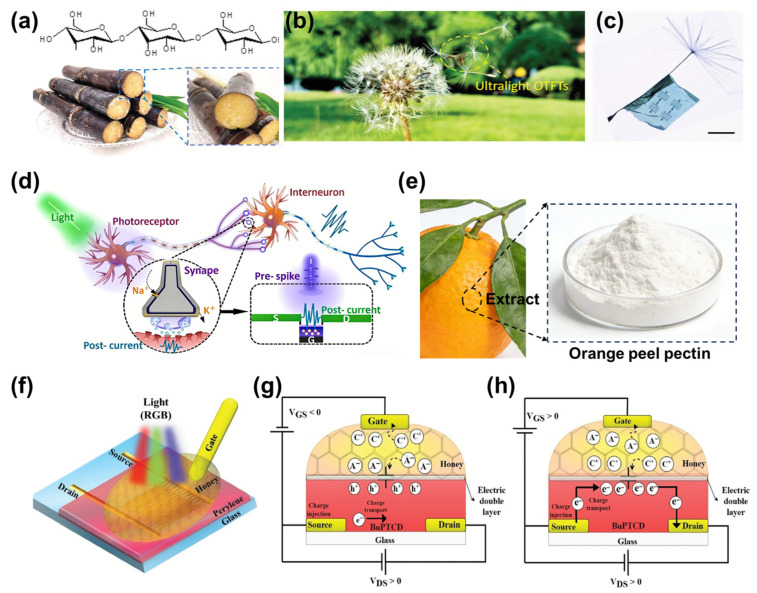
Flexible and degradable organic synaptic transistors based on a dextran dielectric. (**a**) Sugarcane, one of the primary natural sources of dextran, the molecular structure of dextran is shown at the top. (**b**,**c**) Demonstration of the extreme thinness and lightweight nature of the synaptic transistor, enabling it to wrap around a dandelion seed and float freely in air. Scale bar: 300 µm [46]. Copyright 2020, Wiley-VCH GmbH. (**d**) Schematic illustration of photoelectric signal processing in synaptic transistors inspired by biological synapses in the visual neural network. (**e**) Source and molecular structure of the natural acid polyelectrolyte dielectric. (**f**) Three-dimensional schematic illustration of the EGFET. Operating principles of the device under (**g**) negative V_G_ and (**h**) positive V_G_ [16]. Copyright 2025, Wiley-VCH GmbH.

Mechanical flexibility is a defining requirement for wearable, implantable, and bio-integrated neuromorphic vision systems, where devices must operate reliably under continuous deformation and dynamic environments. EGFETs are inherently well suited for such applications due to their soft gel-based electrolytes (e.g., hydrogels and ion gels), low operating voltages, and mechanically compliant material systems. Recent studies have demonstrated that EGFET devices can sustain bending radii as small as a few millimeters and tolerate tensile strains exceeding 10–30%, depending on the substrate and device architecture. Moreover, cyclic bending tests commonly show negligible degradation after 10^3^–10^5^ bending cycles [63], with minimal variation in key parameters such as channel conductance, EPSC, and retention characteristics. These quantitative results highlight the mechanical robustness of electrolyte-gated synaptic devices compared to conventional rigid electronics. The incorporation of bio-derived and biodegradable materials further enhances the sustainability and biocompatibility of such systems [64,65,66]. As shown in Figure 6a, dextran, extracted from renewable natural sources such as sugarcane, serves as an effective dielectric material due to its abundant hydroxyl functional groups and intrinsic ionic conductivity [46]. The molecular structure of dextran enables efficient EDL formation while maintaining excellent mechanical softness and biodegradability. The resulting EGFETs can be fabricated in ultrathin and lightweight formats, leading to extraordinary mechanical compliance. As demonstrated in Figure 6b,c, such ultralight organic synaptic transistors are capable of conformally wrapping around fragile structures, such as dandelion seeds, and even floating freely in air. These visually striking demonstrations highlight the extreme thinness and low bending stiffness achievable with electrolyte-gated architectures, far exceeding the flexibility of conventional solid-dielectric transistors. The intrinsic softness and deformability of gel electrolytes, arising from their three-dimensional polymer networks and high solvent content, play a crucial role in accommodating mechanical strain while maintaining stable ionic transport and interfacial coupling.

Beyond structural flexibility, these devices retain biologically inspired signal-processing functionality. Figure 6d illustrates photoelectric signal processing in synaptic transistors inspired by biological visual neural networks, where optical stimuli are transduced into synaptic currents analogous to pre- and post-synaptic signaling [45]. The combination of mechanical softness and neuromorphic functionality enables robust synaptic operation even under mechanical deformation. In addition to dextran, other natural ionic materials have been explored to further enhance device sustainability. As shown in Figure 6e, natural acid polyelectrolytes derived from plant extracts provide an alternative class of green electrolytes, combining abundant ionic groups with biocompatibility and environmental friendliness. These materials support stable EDL formation and reliable synaptic modulation while reducing reliance on synthetic polymers.

Building upon these bio-derived electrolyte systems, the overall device architecture of flexible synaptic transistors employing natural ionic materials is illustrated in Figure 6f, where Serghiou et al. utilized natural honey as a bio-derived electrolyte dielectric in an EGFET [16]. Owing to its rich ionic constituents and intrinsic proton conductivity, honey enables effective EDL formation at the electrolyte/semiconductor interface. Within this architecture, synaptic behavior originates from the V_G_-controlled redistribution of mobile ions at the interface, leading to dynamic modulation of channel conductance. The operating mechanisms under different V_G_ are further depicted in Figure 6g,h, corresponding to negative and positive gate voltages, respectively. Under these conditions, ion accumulation and depletion induce reversible conductance changes through EDL formation, enabling energy-efficient and controllable synaptic plasticity without involving irreversible chemical reactions. Importantly, EGFET-based synaptic devices constructed from such green and bio-derived materials exhibit outstanding mechanical reliability. Cyclic bending tests reveal negligible degradation in key synaptic parameters, including channel conductance, excitatory postsynaptic current amplitude, and memory retention, even after thousands of deformation cycles. The stable preservation of both short-term and long-term synaptic behaviors under repeated mechanical stress underscores the robustness and practical potential of electrolyte-gated synapses for sustainable and flexible neuromorphic applications [19,67].

Despite these advantages, biodegradable and gel-based electrolytes also present several challenges. First, their high solvent content and soft network structures can lead to limited environmental stability, particularly under varying humidity and temperature conditions, resulting in performance drift over time. Second, ionic conductivity and response speed are often lower or less stable than those of optimized synthetic ion gels, especially in natural materials with heterogeneous compositions. Third, device-to-device reproducibility and large-area uniformity remain challenging, as bio-derived materials may exhibit batch-to-batch variability. Additionally, long-term operation may suffer from ion redistribution, dehydration, or structural degradation, which can affect synaptic reliability and retention. Therefore, future research should focus on improving the stability, uniformity, and controllability of biodegradable electrolyte systems, for example through hybrid material design, crosslinking engineering, and encapsulation strategies. Addressing these limitations will be essential for translating flexible and sustainable neuromorphic devices from laboratory demonstrations to practical applications.

## 6. Challenges and Perspectives

Despite significant progress in flexible electrolyte-gated visual synaptic devices, several critical challenges remain to be addressed before their large-scale and practical implementation. A central issue arises from the intrinsic trade-off between ionic transport dynamics and long-term device stability. Rapid ion migration is essential for achieving fast synaptic responses and high temporal resolution, yet excessive or poorly controlled ionic motion can trigger electrochemical degradation, interfacial instability, and performance drift during prolonged operation [68,69]. This challenge is further exacerbated by the coexistence of optical, electrical, ionic, and mechanical stimuli, which introduces complex multi-physical coupling effects. Such coupled interactions complicate the precise regulation and predictability of synaptic behaviors, particularly under continuous optical excitation or dynamic mechanical deformation. Developing a comprehensive and unified understanding of these intertwined mechanisms remains a fundamental scientific challenge. In addition, device-to-device variability and large-area scalability continue to limit the deployment of high-density neuromorphic vision systems. Variations in material composition, electrolyte distribution, and fabrication processes often lead to non-uniform synaptic characteristics across device arrays, undermining reliable synaptic weight modulation and system-level performance. Addressing these issues will require advances not only in materials synthesis but also in processing control and device architecture standardization [70].

Looking forward, substantial opportunities exist to overcome these challenges through rational materials and device engineering. The development of next-generation electrolytes and semiconductor channels featuring decoupled ionic and electronic transport pathways, improved electrochemical robustness, and intrinsic mechanical compliance holds particular promise for achieving fast, stable, and repeatable synaptic modulation. Interface engineering and architectural optimization will also play a pivotal role in mitigating ion-induced degradation while preserving the hallmark advantages of EGFETs, including ultra-low-voltage operation and high synaptic tunability [13,71]. Beyond device-level improvements, the integration of energy-autonomous functionalities represents an exciting future direction. By coupling flexible visual synapses with energy-harvesting components such as photovoltaic or triboelectric units, it may be possible to realize self-powered neuromorphic vision systems capable of continuous, autonomous operation. More broadly, flexible electrolyte-gated synaptic devices offer a compelling hardware platform for constructing bioinspired visual perception systems that emulate the hierarchical organization of biological vision, spanning photoreception, in-sensor preprocessing, learning, and memory [72]. Leveraging their compatibility with soft materials, low power consumption, and rich plasticity behaviors, these systems could enable transformative applications in artificial intelligence, soft robotics, wearable electronics, and advanced human–machine interfaces, paving the way toward the next generation of intelligent and adaptive visual technologies.

## 7. Conclusions

EGFETs have emerged as a powerful and versatile platform for flexible visual synaptic devices, benefiting from their low-voltage operation, rich and highly tunable synaptic plasticity, and excellent mechanical compliance. Gel-based electrolytes play a pivotal role in enabling high-performance EGFETs by providing efficient ion transport channels, high interfacial capacitance, and excellent mechanical compliance, making them indispensable for next-generation flexible neuromorphic systems. The intrinsic ion–electron coupling mechanism enables dynamic, continuous modulation of channel conductance, allowing EGFETs to faithfully emulate essential synaptic functions of biological visual systems, including both STP and LTP under optical stimulation. Moreover, the broad compatibility of EGFETs with diverse semiconductor channels, electrolyte materials, and flexible substrates facilitates the realization of wearable, conformable, and bio-integrated neuromorphic devices. Beyond individual device performance, EGFET-based visual synapses uniquely support the seamless co-integration of sensing, memory, and information processing within a single hardware unit. This in-sensor computing paradigm substantially reduces system complexity, energy consumption, and signal latency compared with conventional vision architectures, rendering EGFETs particularly attractive for efficient, real-time visual perception. Collectively, these advantages position flexible EGFET synaptic devices as highly promising building blocks for next-generation intelligent perception systems. Looking forward, several key research directions merit further attention. First, improving the long-term stability of gel electrolytes remains critical, particularly in suppressing ion migration-induced degradation and maintaining stable interfacial properties under prolonged operation. Second, a deeper understanding of multi-physical coupling mechanisms, such as ion transport, electronic conduction, and photoexcitation, is essential for achieving precise and predictable synaptic modulation. Third, scalable fabrication strategies and device uniformity across large-area arrays must be addressed to enable practical deployment in integrated systems. In addition, the development of multifunctional gel systems with self-healing, biodegradable, or stimuli-responsive properties could further expand the application scope toward bio-integrated and environmentally sustainable electronics. Finally, the integration of EGFET-based synapses with advanced computing paradigms, such as reservoir computing and hardware neural networks, will be crucial for realizing fully autonomous and intelligent neuromorphic vision systems.

## Figures and Tables

**Table 1:** Representative material systems for electrolyte-gated visual neuromorphic devices.

Semiconductor	Electrolyte	Substrate and Electrode	Synaptic Function	Ref.
P_3_HT-NF	Ion-gel	PDMS, CNTs	PPF, STP	[44]
DNTT	Acidic polyelectrolyte	PEDOT:PSS	PPF, STP, LTP	[45]
DNTT	Dextran	Au	LTP, LTD	[46]
MXene/TAPA	PVA-H_2_SO_4_	Au	LTP	[47]
Perovskite	Polymer electrolyte	PEN, ITO	STP, PPF	[48]
IGZO	Cross-linked electrolyte	PI, ITO	STP, LTP, PPF	[49]
MoS_2_	LiClO_4_/PEO	PMMA	STP	[30]
SnO_2_	LiClO_4_/PMMA	PEN, Au	PPF, STP	[24]
SC-SWCNTs	PVDF-HFP	SEBS, PEDOT:PSS	STP	[50]
MoS_2_	Ionic liquid	PMMA	STP, LPT, PPF	[51]
In_2_O_3_	LiClO_4_/PEO	PI	STP, LTP, PPF	[52]
IGZO	Konjac glucomannan	PET, ITO	PPF, PPD, STP	[29]
InZnSnO nanowires	LiClO_4_/PEO	Mica, Al	STP, LTP	[53]
P_3_HT-NF	Ion-gel	PDMS, AuNP-AgNW	STP, LTP, PPF	[54]

## Data Availability

No new data were created or analyzed in this study.
